# Preoperative nTMS-based mapping of a verbal semantic association task allows for the identification of extensive bihemispheric cortical and subcortical networks

**DOI:** 10.1007/s11060-025-05355-9

**Published:** 2025-12-08

**Authors:** Maximilian Schwendner, Leonie Kram, Haosu Zhang, Sandro M. Krieg, Sebastian Ille

**Affiliations:** Department of Neurosurgery, Heidelberg University Hospital, Ruprecht-Karls-University Heidelberg, Im Neuenheimer Feld 400, 69120 Heidelberg, Germany

**Keywords:** nTMS, Non-invasive brain mapping, Higher cognitive functions, Brain networks, Language mapping

## Abstract

**Purpose:**

Accurately mapping higher cognitive functions remains a challenge both intraoperatively and preoperatively. This study is the first to preoperatively evaluate the bihemispheric cortical and subcortical networks by preoperative nTMS-based mapping of a verbal semantic association task, based on the intraoperatively established Pyramids and Palm Trees Test (PPTT).

**Methods:**

The PPTT was integrated into the established workflow of preoperative nTMS-based mapping. Fibertracking (FT) was performed using a fractional anisotropy (FA) threshold set at 50% of the maximum FA and a minimum fiber length (FL) of 100 mm.

**Results:**

The study included 20 patients with right-sided gliomas. Overall error rates in the lesional (right) hemisphere were 0.108 ± 0.053, compared to 0.098 ± 0.047 in the contralateral (left) hemisphere (*p* = 0.215). Semantic errors were observed more frequently in the right hemisphere (right: 0.0275 ± 0.015, left: 0.016 ± 0.015; *p* = 0.01). Tractography-based network analysis demonstrated comparable network properties regarding fiber volumes (left: 36 ± 16 (14–83) cm^3^, right: 33 ± 15 (9–65) cm^3^; *p* = 0.492), fiber lengths (left: 120 ± 10 (106–140) mm, right: 116 ± 9 (105–144) mm; *p* = 0.185)) and mean FA values (left: 0.37 ± 0.04 (0.32–0.45), right: 0.37 ± 0.04(0.25–0.41); *p* = 0.353) between hemispheres.

**Conclusion:**

The PPTT, when used as a verbal semantic association task, enables a function-based identification of the bihemispheric network underlying semantic association and complex language processing. Function-based FT revealed an extensive right-hemispheric network, comparable in volume, mean FA and fiber length to the left hemisphere. These findings suggest that bihemispheric networks are crucial in higher cognitive functions, including semantic processing during complex language tasks.

**Clinical trial number:**

The trial was registered on clinicaltrials.gov (NCT06401057) on January 7th 2024.

## Introduction

Accurately identifying an individual’s cortical and subcortical networks associated with higher cognitive functions remains challenging, particularly in patients with neurological conditions that enhance brain plasticity, such as glioma.

Navigated transcranial magnetic stimulation (nTMS) is an established noninvasive method for the preoperative identification of eloquent brain areas in patients with brain tumors. nTMS allows to actively interfere with brain functions, modulate brain networks, and mimic “virtual lesions” [1, 2]. In left hemispheric language eloquent brain lesions, nTMS-based language mapping is routinely performed using an object-naming task (ON) [3]. The results derived from preoperative nTMS language mapping show a high correlation with the current gold standard of intraoperative direct cortical stimulation (DCS) [4, 5]. Combined with diffusion tensor imaging, the underlying subcortical networks can be identified by fiber tracking [6, 7]. This data allows for preoperative risk assessment regarding postoperative deficits and enables advanced surgical planning [7, 8] Studies applying nTMS as well as studies with direct cortical stimulation during awake craniotomy for language mapping observed that the error rates correlated with the task performed and with the corresponding task difficulty [9–11]. Apart from ON, tasks like action naming or face naming have already been successfully applied for nTMS mapping [12, 13]. However, the general applicability of the conventional ON task is limited in its applicability. On the one hand, baseline performance is impaired in aphasic patients, and therefore mapping the task is not feasible in severely aphasic patients. On the other hand, it does not cover the extensive brain network of higher cognitive functions linked to language function, such as semantic memory or face recognition, which is also represented in the right hemisphere.

An established test to assess semantic memory is the Pyramids and Palm Trees Test (PPTT), which was first introduced in 1992 by Howard et al. [14]. It is usually applied in patients with cognitive impairments, and especially impaired semantic memory, such as patients with Alzheimer’s disease [14, 15]. However, it is nowadays applied to a broad spectrum of patients, including patients suffering from brain tumors. Especially for advanced brain mapping during awake surgeries, the PPTT is frequently applied [16, 17]. However, intraoperative testing only allows for mapping of the surgical field and is performed in selected cases with eloquent brain areas in peritumoral brain areas.

In this study, we evaluated bihemispheric eloquent brain areas and the underlying subcortical networks involved in semantic association and complex language processing by performing bihemispheric non-invasive nTMS-brain mapping, incorporating the intraoperatively established Pyramids and Palm Trees Test in a verbal response task, and function-based fibertracking.

## Materials and methods

### Patient selection

This study prospectively included patients with primary suspected glioma in the right hemisphere at an eloquent site, undergoing preoperative nTMS examination before microsurgical resection. Patients aged < 18 years, left-handed patients, previous cranial surgery, as well as patients with general magnetic resonance imaging (MRI) and nTMS exclusion criteria such as cochlear implants or cardiac pacemakers were excluded from this study.

### Preoperative imaging

Preoperative MRI acquisition was performed according to clinical routine. All patients obtained a structural MRI scan (3 Tesla magnetic resonance scanner) according to the standard MRI protocol at our institution, including a three-dimensional gradient echo sequence with intravenous contrast administration and diffusion tensor imaging (DTI) sequence with 16 directions before surgery.

### nTMS mapping

Testing was performed according to the clinically established workflow, using a Nexstim eXSima NBS 5.2.4 device (Nexstim Plc, Helsinki, Finland).

Stimulation sites were predefined by Corina’s cortical parcellation system including 21 brain regions with 46 stimulation sites on each hemisphere, according to clinical routine [11]. Each stimulation site was stimulated three times. Stimulation was performed as repetitive stimulation with 10 stimuli of a frequency of 5 Hz with an intensity of 110% of the resting motor threshold on both hemispheres.

Synchronized with nrTMS, the PPTT task was presented. The PPTT test set consists of a test set of 52 individual items and is an established test to be assessed during awake surgery [18]. All items were integrated into to picture presentation software NexSpeech^®^ 2.1.0 (Nexstim Plc, Helsinki, Finland), simultaneously recording video and audio of the responses to the tasks presented for post hoc analysis. Testing was performed using an inter-picture-interval (IPI) of 4500–5000 msec adapted to the patient’s baseline with a picture presentation time (PPT) of 2500msec and a picture-to-trigger interval (PTI) of 0 msec.

A baseline without nTMS was performed prior to nTMS testing, showing all image sets twice. Only images with a consistent and correct performance were used. A cut-off of 50% (26 image sets) was required to perform nTMS testing.

### nTMS data analysis

After nTMS-mapping, all stimulations including the corresponding recordings of video and audio were reviewed post-hoc by a language therapist experienced in awake surgeries, using NexSpeech Analyzer 2.10 (Nexstim Plc, Helsinki, Finland).

Errors were classified as no response, including hesitation, performance errors, including articulatory errors, semantic errors, including errors in word order, and phonological paraphasia.

### nTMS based fiber tracking

Function-based fiber tracking was applied using diffusion tensor imaging (DTI) and a deterministic fiber tracking algorithm. Fiber tracking modalities were adapted from function-based fiber tracking of language function, which has already been validated with clinical outcomes in both low-grade and high-grade glioma of the left hemisphere [19, 8]. A minimum fiber length of 100 mm and a maximum angulation of 20° were applied. Regarding fractional anisotropy (FA), we performed fiber tracking according to clinical routine at a fractional anisotropy threshold of 50% of the maximum FA, as well as at a fixed FA threshold of 0.10 for better comparability. nTMS-based cortical data with a margin of 5 mm were used as regions of interest for function-based fiber tracking.

### Data analysis

Statistical analyses were performed using Prism (version 9.1.1; GraphPad Software, La Jolla, CA, USA). Descriptive statistics were calculated for the patient- and tumor-related characteristics, including mean, median, minimum, maximum, and standard deviation. Comparisons between both groups were performed using the Wilcoxon matched-pairs signed rank test. The level of significance was set at *p* < 0.05.

## Results

### Patient and tumor characteristics

Twenty cases suffering from glioma scheduled for microsurgical resection successfully underwent preoperative nTMS mapping of the PPTT task and were therefore included in this study. The mean age was 54.4 ± 16.1 (27.4–78.2) years and patients were predominantly female (11 patients; 55.0%). The most frequent lesion sites were frontal (35.0%) and temporal (30.0%) (Table 1). Mean tumor volume was 33.9 ± 22.9 (3.5–78.3) cm^3^. Histopathological findings showed high-grade glioma in 15 cases (75.0%) and low-grade glioma in 5 cases (25.0%) (Table 1).

**Table 1:** Illustrates patient and tumor characteristics of all 20 cases with right-sided lesions included in this study

Age (years) (Mean ± SD (Min-Max))	54.4 ± 16.1 (27.4–78.2)
**Gender** • Female n(%)) • Male (n(%))	11 (55.0%) 9 (45.0%)
**Handedness** • Right-handed (n(%))	20 (100%)
**Histopathological findings** • Low-grade glioma (n(%)) • High-grade glioma (n(%))	5 (25.0%) 15 (75.0%)
**Side** • Right (n(%))	20 (100%)
**Brain region** • Frontal (n(%)) • Temporal (n(%)) • Parietal (n(%)) • Insular (n(%))	7 (35.0%) 6 (30.0%) 4 (20.0%) 3 (15.0%)

### nTMS mapping of the PPTT task

Preoperative nTMS-mapping of the PPTT task was successfully performed in all twenty cases. Overall, errors occurred on the right hemisphere in 0.108 ± 0.053 (0.029–0.225) of all stimulation sites and on the left in 0.098 ± 0.047(0.036–0.167) (*p* = 0.215) (Table 2).

**Table 2:** Cortical distribution of error rates

CPS region	Right hem. All errors	Left hem. All errors	Right hem. Semantic	Left hem. Semantic
AnG	0.121	0.096	0.025	0.013
aSMG	0.092	0.100	0.042	0.008
aSTG	0.117	0.133	0.050	0.000
dPoG	0.067	0.083	0.000	0.000
dPrG	0.083	0.133	0.017	0.050
mMFG	0.119	0.100	0.036	0.022
mMTG	0.075	0.058	0.017	0.008
mPoG	0.100	0.108	0.025	0.025
mPrG	0.175	0.108	0.050	0.042
mSFG	0.111	0.078	0.033	0.011
mSTG	0.058	0.108	0.033	0.033
opIFG	0.183	0.100	0.050	0.017
pMFG	0.100	0.150	0.025	0.017
pMTG	0.044	0.094	0.011	0.011
pSFG	0.117	0.050	0.000	0.017
pSMG	0.150	0.133	0.025	0.008
pSTG	0.117	0.133	0.017	0.017
SPL	0.108	0.042	0.017	0.000
trIFG	0.094	0.044	0.033	0.006
vPoG	0.125	0.108	0.017	0.033
vPrG	0.100	0.150	0.017	0.008
Overall	0.108	0.098	0.028	0.016

Table 2 outlines the error rates for all individual brain regions according to the cortical parcellation system (CPS) for the Pyramid and Palm Trees Test (PPTT), used as a verbal semantic association task, during navigated transcranial magnetic stimulation (nTMS). It presents the error rates across all error categories and only for semantic errors (semantic) for the right hemisphere (right hem.) and left hemisphere (left hem.)

AnG: Angular gyrus; aSMG: Anterior supramarginal gyrus; aSTG: Anterior superior temporal gyrus; dPoG: Dorsal postcentral gyrus; dPrG: Dorsal precentral gyrus; mMFG: Middle middle frontal gyrus; mMTG: Middle middle temporal gyrus; mPoG: Middle postcentral gyrus; mPrG: Middle precentral gyrus; mSFG: Middle superior frontal gyrus; mSTG: Middle superior temporal gyrus; opIFG: Opercular part of the inferior frontal gyrus; pMFG: Posterior middle frontal gyrus; pMTG: Posterior middle temporal gyrus; pSFG: Posterior superior frontal gyrus; pSMG: Posterior supramarginal gyrus; pSTG: Posterior superior temporal gyrus; SPL: Superior parietal lobule; trIFG: Triangular part of the inferior frontal gyrus; vPoG: Ventral postcentral gyrus; vPrG: Ventral precentral gyrus; Overall: Combined error rates

**Fig. 1:**
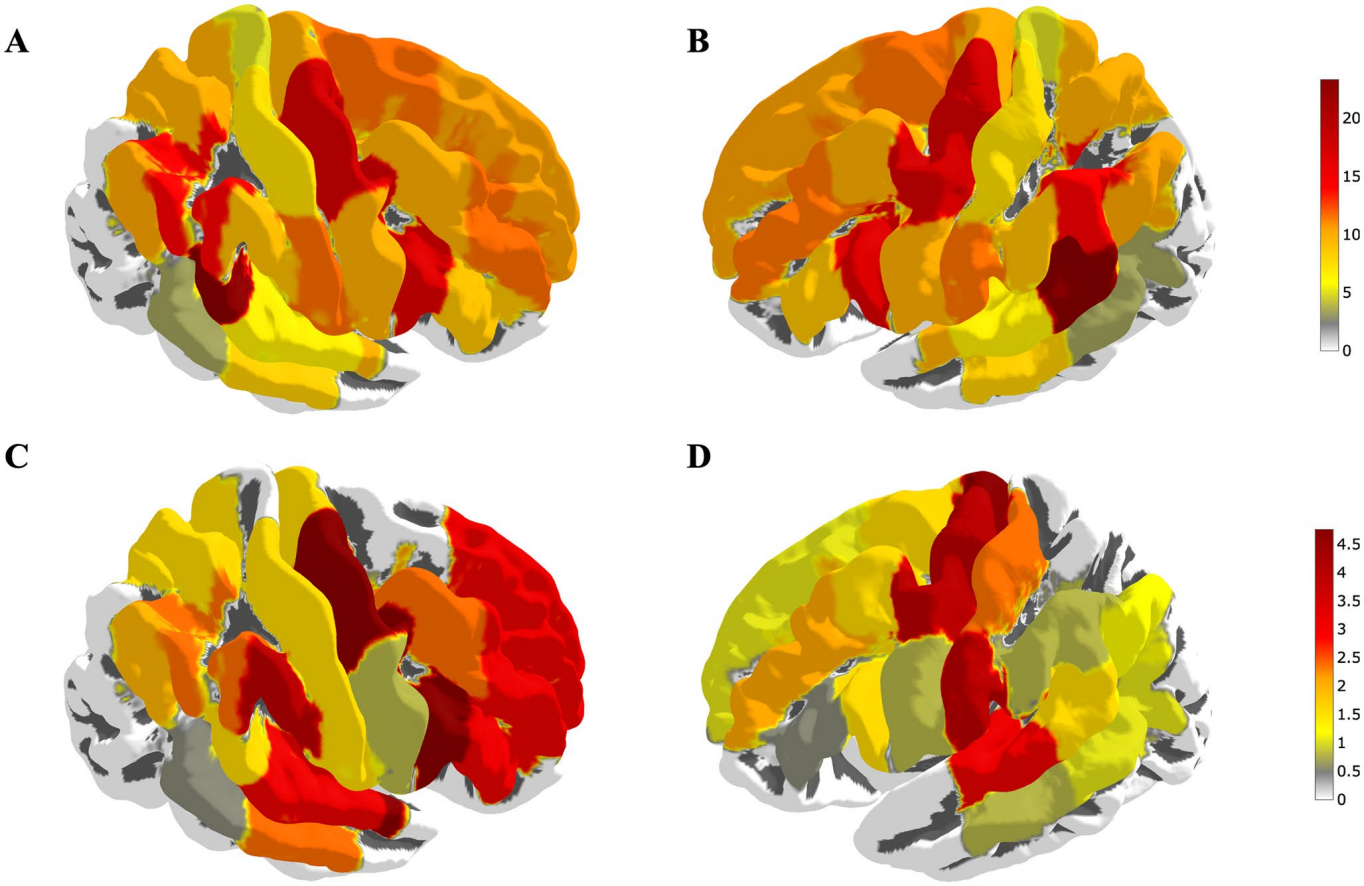
Cortical distribution of error rates. Figure 1 depicts the distribution of error rates for the Pyramid and Palm Trees Test (PPTT), used as a verbal semantic association task during navigated transcranial magnetic stimulation (nTMS). It presents the error rates across all error categories (**A**, **B**) and only for semantic errors (**C**, **D**)

Regarding overall error rates, the most frequently positively mapped CPS regions in the right hemisphere were opIFG (18.3%), mPrG (17.5%), pSMG (15.0%), and vPoG(12.5%). In the left hemisphere, it was pMFG (15.0%), vPrG(15.0%), dPrG (13.0%), aSTG (13.0%), pSTG (13.0%) and pSMG (13.0%) (Table 2; Fig. 1).

Semantic errors were observed on the right hemisphere in 0.0275 ± 0.015 (0.00-0.058) of stimulations and on the left hemisphere in 0.016 ± 0.015 (0.00-0.051) (*p* = 0.011). On the right, lesional hemisphere, semantic errors were most frequently observed in aSTG (5.0%), mPrG (5.0%), and opIFG (5.0%). For the left hemisphere, the highest rates of semantic errors were observed in dPrG (5.0%), mPrG (4.2%), mSTG (3.3%), and vPoG (3.3%) (Table 2).

### Function-based fiber tracking using the PPTT task

**Fig. 2:**
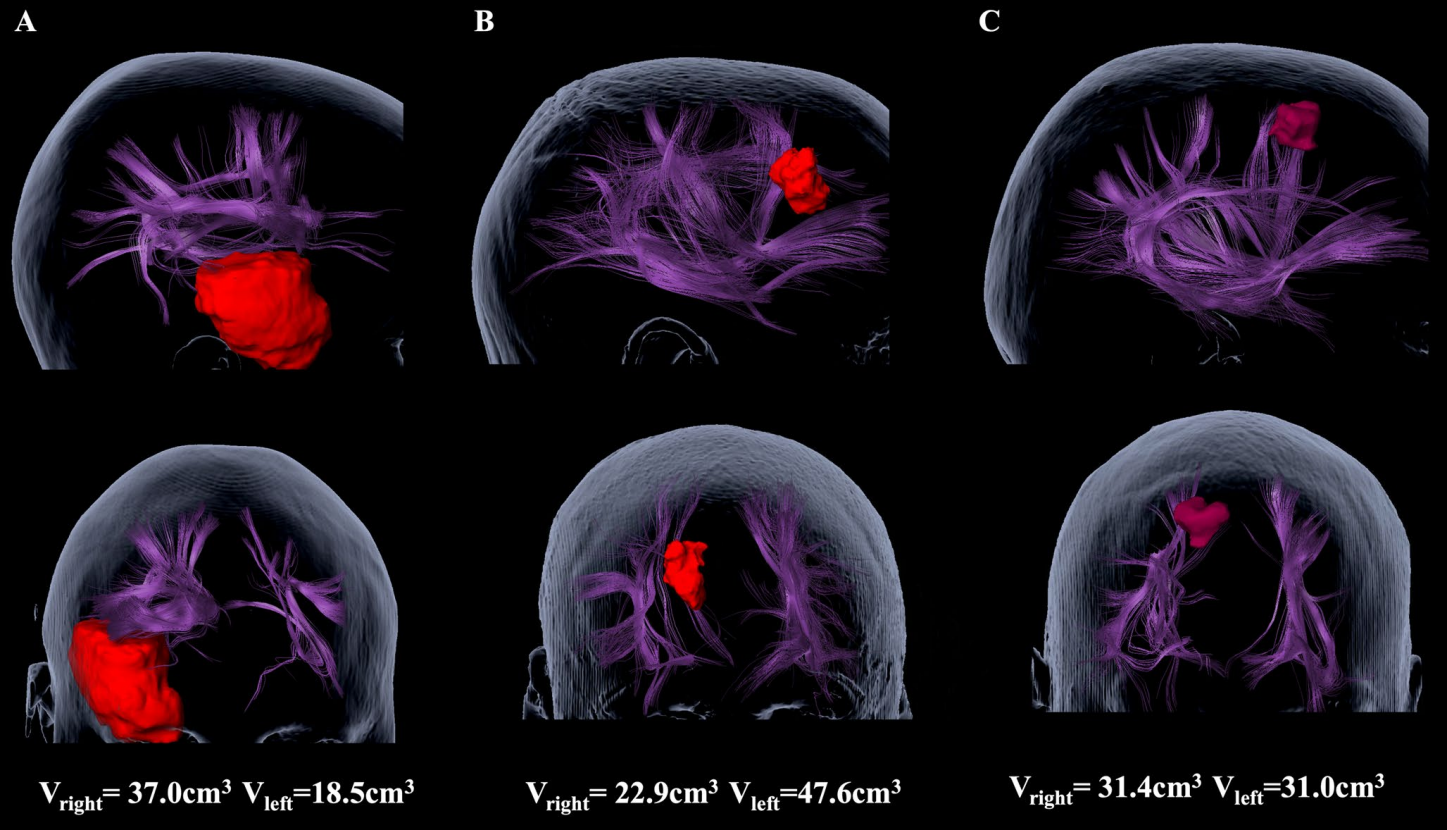
Subcortical analysis in exemplary patient cases. In Figure **A**, a patient with an extensive lesion in the anterior temporal lobe is shown, with particularly larger fiber volumes on the right hemisphere (V_right_) compared to the left hemisphere (V_left_), especially for the SLF II, III, and AF. In Figure **B**, a lesion in the frontal lobe affecting SLF II, III and AF with a therefore lower fiber volume is illustrated. In Figure **C**, balanced volumes between both hemispheres were observed, with a small lesion in the SFG

Function-based fibertracking, using nTMS positive stimulation sites as seeding ROIs was successfully performed in all 20 patients with an FA of 0.50 of the maximum FA (FA_max_) and a FA of 0.10 (Fig. 2).

**Fig. 3:**
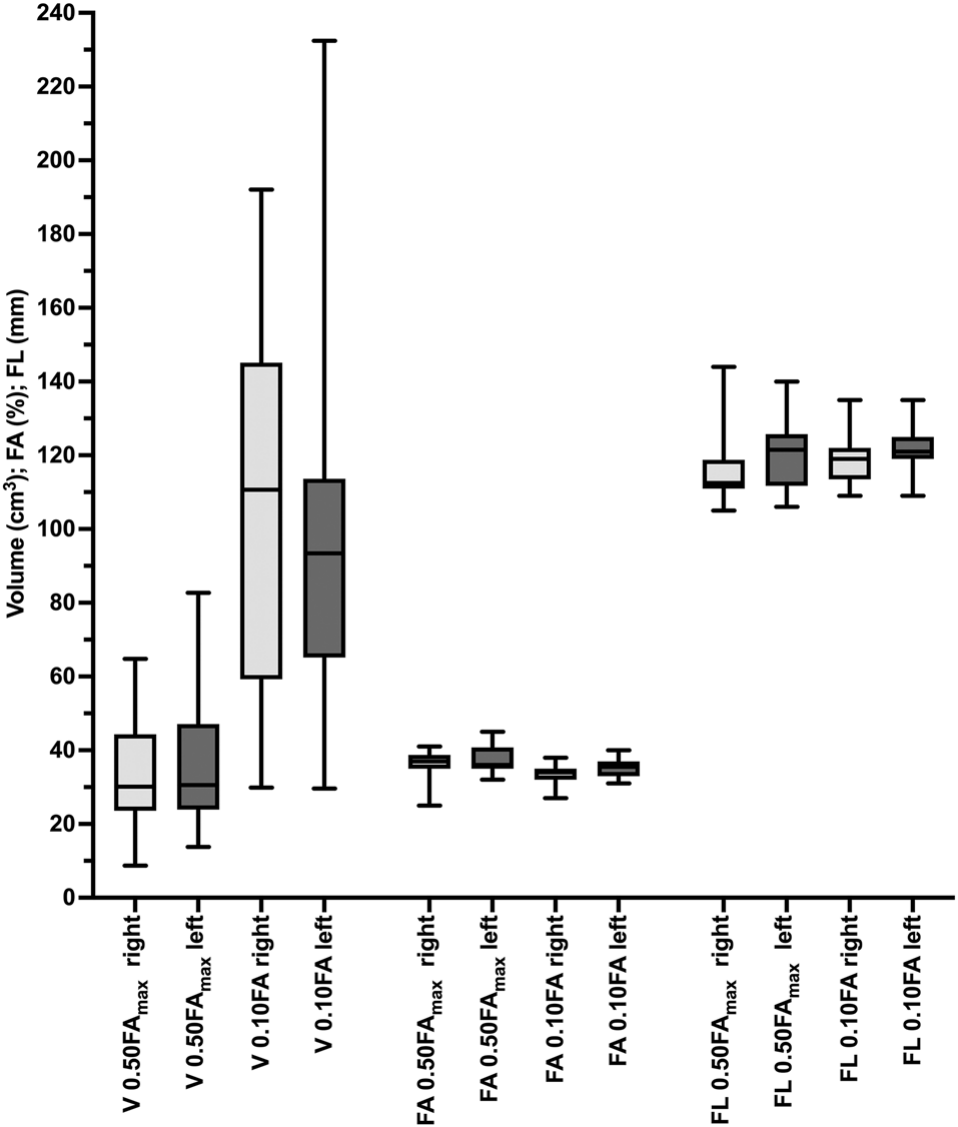
Analysis of function-based subcortical networks. Figure 3 compares network modalities of the subcortical networks for the right and left hemispheres as identified by function-based fibertracking at a fractional anisotropy of 0.50 of the maximum FA (0.50FA_max_) and at a FA of 0.10 (0.10FA). Fiber volumes (V), fiber length (FL) and fractional anisotropy (FA) were compared

Comparing the networks of the right and left hemispheres, we observed comparable network properties. For a FA of 0.50 FA_max_, the mean FA in the right hemisphere FA_right_ was 0.37 ± 0.04 (0.25–0.41), compared to a mean FA of the left hemisphere (FA_left_) of 0.37 ± 0.04 (0.32–0.45) (*p* = 0.353) (Fig. 3). Network volumes were comparable between hemispheres with a mean volume of the right hemisphere of V_right_ 33 ± 15 (9–65) and of the left hemisphere of V_left_ 36 ± 16 (14–83) (*p* = 0.492). Mean fiber lengths were similar for the left hemisphere (FL_left_ = 120 ± 10 (106–140)) and right hemisphere (FL_right_ =116 ± 9 (105–144)) (*p* = 0.185) (Fig. 3).

For fibertracking at an FA of 0.10, we observed a significantly higher FA in the network of the left hemisphere with an FA_leftt_ of 0.35 ± 0.03 (0.31–0.40) and an FA_right_ of 0.34 ± 0.03(0.27–0.38) (*p* = 0.004). Network volumes were comparable with V_right_ 108 ± 50 (30–192) and V_left_ 96 ± 46 (30–232) (*p* = 0.202), as well as fiber lengths with FL_right_ = 120 ± 7 (109–135) and FL_left_ =121 ± 7 (109–135) (*p* = 0.236).

## Discussion

### Summary of results

For the first time, we conducted non-invasive preoperative mapping of advanced brain functions including semantic memory in a structured manner for the whole hemisphere of both sides in a cohort of patients suffering from right-sided gliomas.

Analysis did not only reveal comparable error rates of both hemispheres on a cortical level, but also showed comparable subcortical networks regarding the network properties mean FA, mean fiber length and network volume.

### Clinical application of the PPTT

The PPTT is a semantic memory test that measures the capacity to access detailed semantic information on words and pictures to identify the underlying analogies [14]. The test in its original form is a nonverbal semantic test without a given time limit per task and was initially used to test patients with dementia, such as Alzheimer’s disease or primary progressive aphasia [15, 20, 21]. It is nowadays an established test applied during awake surgeries, including modified forms, such as combinations with verbal naming of the objects of the task [18, 22, 23].

### Brain areas linked to semantic processing and reasoning

The PPTT is a cognitive test that evaluates semantic processing. Incorporating the test as a verbal response task within a limited response time frame effectively increases task complexity. The test necessitates the use of semantic memory, visual perception, visuospatial attention, language production, and articulatory planning.

The inclusion of short-term memory by testing each task twice during the baseline further increases the number of potentially involved brain regions.

Regarding the distribution of cortical brain areas related to semantic tasks, core linguistic functions, e.g., syntax, can be found primarily in the left frontotemporal system, whereas more general communicative abilities, e.g., semantics, are related to a bilateral frontotemporal system [24]. Wright et al. performed structural and functional fMRI analysis in patients with chronic left hemispheric lesions and healthy controls [24]. The authors reported that semantic performance correlated with activity in the right superior and middle temporal gyri regardless of tissue integrity [24]. As the activity in the right temporal brain areas did not differ between patients and controls, the authors suggest that the semantic network is redundantly organized and regions in both hemispheres can perform similar tasks [24].

The anterior temporal lobe plays a major role in semantic processing. Rice et al. showed the importance of the left and right anterior temporal lobe in semantic memory in a cohort of patients undergoing anterior temporal lobectomy [25]. The left anterior temporal lobe shows a more prominent role in word retrieval, whereas both, the left and right anterior temporal lobe contribute to semantic processing [25]. Pobric et al. applied continuous low-frequency rTMS of 1 Hz over 10 min to the left anterior temporal lobe, observing a selective semantic impairment [26].

Furthermore, differences were additionally observed between abstract and concrete semantic processing. Garcia et al. analyzed the functional connectivity of brain networks using an fMRI connectivity analysis for three seeds in the left cortex (aMTG, AG, and IFG) during abstract and concrete semantic processing [27]. Comparing both tasks, greater bilateral connectivity from the left AG to frontal, parietal, occipital, and anterior cingulate clusters was observed during abstract semantic processing [27]. This was also evident between the left aMTG and parietal and occipital clusters [27]. The IFG had more extensive connectivity with cortical areas of the left hemisphere during concrete processing, whereas the connectivity to areas of the right hemisphere did not differ significantly between the semantic conditions [27].

Regarding the component of reasoning, a variety of brain areas are activated during inductive and deductive reasoning processes. Shin et al. conducted a cortical surface-based meta-analysis to reveal areas commonly activated in inductive and deductive reasoning processes [28]. In the left hemisphere, activations in the dorsolateral prefrontal, inferior frontal cortex, orbital and polar frontal cortex, anterior cingulate and medial prefrontal cortex, insular and frontal opercular cortex, and paracentral lobular and midcingulate cortex were observed [28, 29]. The corresponding activations in the right hemisphere were smaller than those in the left hemisphere and found in the dorsolateral prefrontal cortex, inferior and superior parietal cortex, anterior cingulate and medial prefrontal cortex, and paracentral lobular and mid-cingulate cortex [28, 29].

When considering the impact of the right hemisphere on cognitive and language-related tasks, Gajardo-Vidal et al. analyzed language impairments in patients suffering from right-hemispheric stroke [30]. In patients with unilateral right-hemisphere damage, the highest incidence of impaired performance was recorded for an auditory sentence-to-picture matching task with significant activation in the right inferior frontal sulcus and right mediodorsal thalamus on fMRI, which were also responsive to a wide range of language tasks that taxed phonological, semantic, sentence and verbal short-term memory processing [30]. Errors in a picture-to-picture matching task, similar to the PPTT, were predominantly observed in patients with visuospatial impairments and were otherwise comparable between patients with left-sided and right-sided strokes [30].

### Integrating verbal tasks into nTMS testing

In this setting, the PPTT task was modified, combining the semantic memory task with the corresponding object naming task. Furthermore, testing was performed for a selected set of items, which were performed correctly twice during the baseline acquisition and within a time limit of 4500 to 5000 msec.

As all error-prone PPTT stimuli were removed during the baseline testing in a standardized manner, the high error rate of the PPTT cannot be solely explained by this task being more error-prone in general.

More difficult tasks generally require larger functional networks. The higher cognitive effort needed to perform the PPTT task is likely to be more impaired by rnTMS creating a virtual lesion, which may result in the higher error rate observed in this study compared to the Object naming task. This might be due to the recruitment of larger networks, being more susceptible to modulation at more cortical sites or stimulation at lower intensities.

Higher error rates in more complex tasks were also observed by Ohlerth et al., investigated nTMS-based mapping of the object naming task and action naming task in a cohort of 20 healthy volunteers [10]. In both hemispheres, higher error rates were found in the action naming task compared to the object naming task. Action naming showed higher error rates across the entire hemisphere, not limited to particular cortical brain areas [10].

### Intraoperative testing of higher brain functions

Intraoperative cortical mapping using direct cortical stimulation and an object-naming task showed a widespread distribution across perisylvian brain areas and areas around the central sulcus, but particularly error-prone areas were cortical regions in the superior and medial temporal gyrus, postcentral gyrus, and supramarginal gyrus for semantic errors [11, 31]. No-response errors were observed especially in the posterior frontal gyri and precentral gyrus, while also including the superior and medial temporal gyrus, postcentral gyrus, and supramarginal gyrus [11, 31]. nTMS-based mapping of the modified PPTT task in this study also resulted in high error rates in these brain areas on the left, non-lesional hemisphere. (Table 1).

The PPTT can also be applied in intraoperative testing during cortical and subcortical stimulation in awake craniotomies for glioma resection as a nonverbal test for semantic processing or as a task of combined picture naming and retrieval of comprehensive semantic information [23, 32].

In a study performed by Chang et al. PPTT testing during subcortical stimulation in awake glioma surgery showed a superior correlation with postoperative outcome compared to an object naming task, when using the PPTT task with verbal responses, combining picture naming and the retrieval of comprehensive semantic information [23].

Regarding bilateral networks, Cuisenier et al. performed DCS and task-induced high-frequency activity recording to evaluate the bilateral distribution of language function in forty-two patients before epilepsy surgery [33]. Induced high-frequency activity analysis revealed a larger frontotemporoparietal bilateral language network in contrast to the left-restricted frontotemporal language network identified by DCS [33]. Direct cortical stimulation of the right hemisphere in patients with right-sided low-grade glioma revealed language-eloquent cortical brain areas in the precentral gyrus, ventral premotor cortex, postcentral gyrus, and superior temporal gyrus [34]. Similar findings were observed in our study (Fig. 1).

A study on awake surgery for gliomas involving the right inferior parietal lobe in 14 patients highlighted the role of the PPTT task, revealing that nonverbal semantic disorders were induced in 7 patients when white matter connections of the right inferior frontooccipital fascicle were stimulated [35]. However, on the cortical surface, no sites were found to induce disturbances in PPTT task performance [35].

A study by Herbet et al. analyzed findings of awake surgeries in 13 patients with right low-grade glioma located within or close to the ventral stream, observing non-verbal semantic-related cortical sites in 6 patients in structures commonly associated with verbal semantic processing in the left hemisphere, including the superior temporal gyrus, the pars triangularis, and the dorsolateral prefrontal cortex [36]. At the subcortical level, the authors found non-verbal semantic-related sites in all but one patient, with all responsive stimulation points located on the spatial course of the right inferior frontooccipital fasciculus, providing further evidence for a critical role of the right IFOF in non-verbal semantic processing [36].

When testing the PPTT task as a nonverbal semantic in the right frontal lobe during awake surgery in glioma patients, Barberis et al. reported no reproducible stimulation sites [37].

### Clinical implications

The present study demonstrates the feasibility of incorporating the PPTT as a verbal task into nTMS mapping to delineate expansive brain networks within the right hemisphere. Our findings, particularly regarding the cortical distribution of eloquent brain regions identified through nTMS, exhibit a strong concordance with existing literature, including data derived from direct cortical stimulation.

Preoperative nTMS mapping utilizing the PPTT task warrants consideration, particularly for addressing deep-seated right-sided lesions that are typically excluded from awake surgical protocols in many institutions.

### Limitations

This study presents findings from a cohort of 20 patients all diagnosed with right-hemispheric gliomas.

Due to the limited sample size, data could not be adequately adjusted for potential confounders such as lesion location, tumor volume, or the specific cognitive domains affected. Although a stable baseline performance in the PPTT was required for all patients, residual variability in cognitive capacity cannot be fully excluded.

As this study aimed to provide a comprehensive analysis across the entire cortical surface and both hemispheres in a cohort only undergoing awake surgery in selected cases, comparative data from established methods, such as direct cortical and subcortical stimulation, were not available for this patient cohort.

Additional limitations arise at the subcortical level, primarily due to the methodological constraints of function-based deterministic fiber tracking. This technique delineates subcortical pathways based on cortical sites identified as eloquent by the PPTT, a task that does not encompass the full breadth of higher cognitive functions. Consequently, the identified network might be limited to fiber tracts engaged by the semantic association task. Furthermore, fiber tracking was performed in both the lesional and non-lesional hemispheres, which may limit the generalizability of the findings—both regarding hemispheric comparisons and across different tumor grades and entities.

## Conclusion

The PPTT, when used as a verbal semantic association task, enables function-based identification of the bihemispheric networks underlying semantic association and complex language processing. The task can be easily integrated into the workflow of nTMS language mapping.

On a cortical level, non-invasive brain mapping testing the PPTT tasks resulted in comparable error rates between both hemispheres and showed a distribution pattern of eloquent brain areas comparable to intraoperative findings from direct cortical stimulation.

Function-based fibertracking of the PPTT task revealed an extensive network in the right hemisphere, comparable to the left hemisphere in network properties.

These findings indicate that bihemispheric networks contribute substantially to higher cognitive functions, including semantic processing during language tasks. However, given the limited sample size and the inability to fully control for relevant confounding variables, these results should be interpreted as exploratory. Larger, controlled studies are needed to confirm and refine these observations.

## Data Availability

In the interest of patient privacy, all the collected raw data of individual cases is imparticipable. The anonymous datasets used and analyzed during the current study, the study protocol, and the statistical analysis plan are available up on reasonable request from the corresponding author to researchers who provide a methodologically sound proposal beginning 3 months and ending 5 years following article publication. Proposals should be directed to [Maximilian.Schwendner@med.uni-heidelberg.de](mailto: Maximilian.Schwendner@med.uni-heidelberg.de) ; to gain access, data requestors will need to sign a data access agreement.

## Abbreviations

anG: Angular gyrus
aSMG: Anterior supramarginal gyrus
aSTG: Anterior superior temporal gyrus
dPoG: Dorsal postcentral
dPrG: Dorsal precentral gyrus
mMFG: Middle middle frontal gyrus
mMTG: Middle middle temporal gyrus
mPoG: Middle postcentral gyrus
mPrG: Middle precentral gyrus
MRI: Magnetic resonance imaging
mSFG: Middle superior frontal gyrus
mSTG: Middle superior temporal gyrus
nTMS: Navigated transcranial magnetic stimulation
opIFG: Opercular inferior frontal gyrus
pMFG: Posterior middle frontal gyrus
pMTG: Posterior middle temporal gyrus
pSFG: Posterior superior frontal gyrus
pSMG: Posterior supramarginal gyrus
pSTG: Posterior superior temporal gyrus
rnTMS: Repetitive navigated transcranial magnetic stimulation
SPL: Superior parietal lobe
trIFG: Triangular inferior frontal gyrus
vPoG: Ventral postcentral gyrus
MRI: Magnetic resonance imaging
